# Chromosome 7 and 19 Trisomy in Cultured Human Neural Progenitor Cells

**DOI:** 10.1371/journal.pone.0007630

**Published:** 2009-10-29

**Authors:** Dhruv Sareen, Erin McMillan, Allison D. Ebert, Brandon C. Shelley, Julie A. Johnson, Lorraine F. Meisner, Clive N. Svendsen

**Affiliations:** 1 Department of Neurology, University of Wisconsin School of Medicine and Public Health, Wisconsin Institutes for Medical Research (WIMR), Madison, Wisconsin, United States of America; 2 The Stem Cell and Regenerative Medicine Center, University of Wisconsin, Madison, Wisconsin, United States of America; 3 The Waisman Center, University of Wisconsin, Madison, Wisconsin, United States of America; 4 Cell Line Genetics, LLC, Madison, Wisconsin, United States of America; Universidade Federal do Rio de Janeiro (UFRJ), Instituto de Biofísica da UFRJ, Brazil

## Abstract

**Background:**

Stem cell expansion and differentiation is the foundation of emerging cell therapy technologies. The potential applications of human neural progenitor cells (hNPCs) are wide ranging, but a normal cytogenetic profile is important to avoid the risk of tumor formation in clinical trials. FDA approved clinical trials are being planned and conducted for hNPC transplantation into the brain or spinal cord for various neurodegenerative disorders. Although human embryonic stem cells (hESCs) are known to show recurrent chromosomal abnormalities involving 12 and 17, no studies have revealed chromosomal abnormalities in cultured hNPCs. Therefore, we investigated frequently occurring chromosomal abnormalities in 21 independent fetal-derived hNPC lines and the possible mechanisms triggering such aberrations.

**Methods and Findings:**

While most hNPC lines were karyotypically normal, G-band karyotyping and fluorescent *in situ* hybridization (FISH) analyses revealed the emergence of trisomy 7 (hNPC^+7^) and trisomy 19 (hNPC^+19^), in 24% and 5% of the lines, respectively. Once detected, subsequent passaging revealed emerging dominance of trisomy hNPCs. DNA microarray and immunoblotting analyses demonstrate epidermal growth factor receptor (EGFR) overexpression in hNPC^+7^ and hNPC^+19^ cells. We observed greater levels of telomerase (hTERT), increased proliferation (Ki67), survival (TUNEL), and neurogenesis (β_III_-tubulin) in hNPC^+7^ and hNPC^+19^, using respective immunocytochemical markers. However, the trisomy lines underwent replicative senescence after 50–60 population doublings and never showed neoplastic changes. Although hNPC^+7^ and hNPC^+19^ survived better after xenotransplantation into the rat striatum, they did not form malignant tumors. Finally, EGF deprivation triggered a selection of trisomy 7 cells in a diploid hNPC line.

**Conclusions:**

We report that hNPCs are susceptible to accumulation of chromosome 7 and 19 trisomy in long-term cell culture. These results suggest that micro-environmental cues are powerful factors in the selection of specific hNPC aneuploidies, with trisomy of chromosome 7 being the most common. Given that a number of stem cell based clinical trials are being conducted or planned in USA and a recent report in PLoS Medicine showing the dangers of grafting an inordinate number of cells, these data substantiate the need for careful cytogenetic evaluation of hNPCs (fetal or hESC-derived) before their use in clinical or basic science applications.

## Introduction

Stem cell expansion and differentiation is the foundation of emerging cell therapy technologies. The most primitive embryonic stem cells (ESCs), derived from the developing blastocyst, have diploid karyotypes that can remain stable after many passages *in vitro* [1], [2]. However, there have been many reports that these cells may acquire specific recurrent chromosomal abnormalities after prolonged culture. These include aneuploidy with gain of chromosomes (trisomy) 12, 17, and X in human ESCs [2]–[5] and trisomy 8 and 11 in mouse ESCs [6], [7]. These abnormalities lead to differential growth rates [2]–[4], thus lessening the reproducibility and reliability. Furthermore, use of hESCs for clinical trials must ensure that they maintain a normal karyotype to avoid possible malignant tumor formation after transplantation.

More restricted human neural progenitor cells (hNPCs) can be isolated from different regions of the developing human brain, expanded in culture and then differentiated into neurons and astrocytes [8]–[12]. There have been many reports showing that hNPCs can be used *in vitro* to study the mechanisms of neurogenesis and also transplanted into the developing or adult rodent brain [8]–[12]. Upon grafting, the cells have the ability to integrate, migrate and develop into both neurons and astrocytes [13]–[15] without any evidence of teratoma formation, a common problem for studies using hESCs. As such they represent an interesting source of tissue for cell therapy, either alone or following modification to release potent growth factors [16], [17].

Compared to hESCs, most hNPCs do not express high levels of telomerase and show senescence patterns after between 50 and 70 population doublings [18], [19]. There are suggestions that telencephalic proliferative regions of the mammalian brain, including the embryonic cerebral cortex and postnatal SVZ may contain a population of aneuploid cells [20]–[22]. Nevertheless, there have been no previous reports of recurrent chromosome changes in cultures of hNPCs. In the current study, after karyotyping 21 hNPC lines in preparation for generating a clinically viable cell bank, we discovered for the first time that trisomy 7 occurs in five independently derived hNPC lines and trisomy 19 was observed in only one hNPC line. Once established, the lines with trisomy have a selective advantage. One of the most susceptible lines showed high levels of endogenous telomerase expression in the trisomy hNPCs, and stress through epidermal growth factor (EGF) deprivation potentially triggered selection and enrichment of trisomy 7 cells in an otherwise normal line.

## Results

### Gain of chromosome 7 or 19 can be found in some hNPC lines

**Figure 1:**
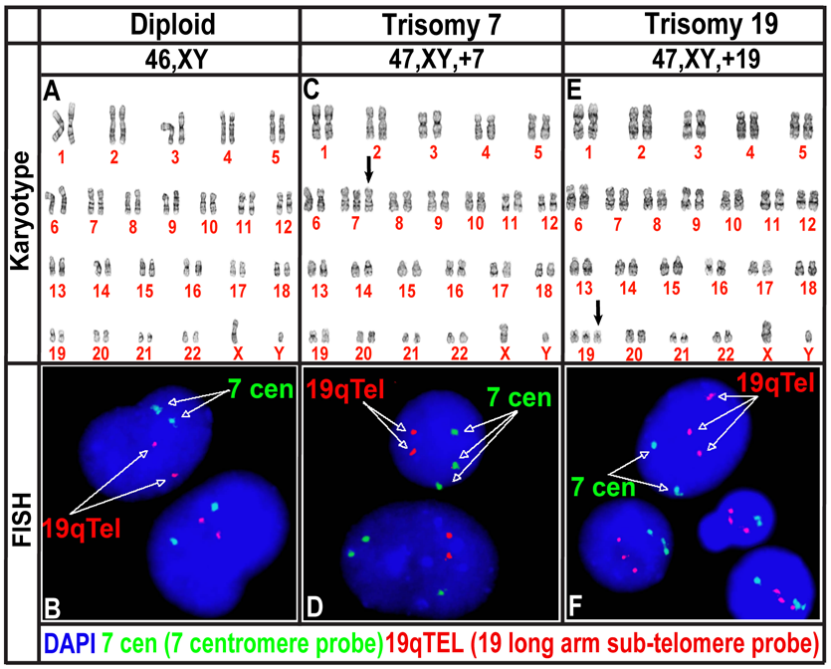
Trisomy of chromosome 7 and 19 in M031 CTX hNPC line. (A, B) Compared to the wild type controls (^dip^), (C, D) sub-cultures of M031 CTX line display a complete trisomy of chromosome 7 (^+7^), (E, F) and a trisomy of chromsome19 (^+19^) after fourteen to thirty-eight passages. (A, C, E) Twenty metaphase cells were examined by G-banding, (B, D, F) and 200 interphase nuclei were evaluated by fluorescence in-situ hybridization (FISH). Results are representative of at least one of three independent biological samples with similar results.

Human NPCs isolated from fetal brain tissue between ten to fifteen weeks of post-conception were maintained in neural expansion medium consisting of epidermal growth factor (EGF) and fibroblast growth factor-2 (FGF-2) and then switched to EGF and leukemia inhibitory factor (LIF) as described in detail previously [19], [23]. Twenty-one independent lines of hNPCs derived from cortex or ventral mesencephalon samples, were cultured, karyotyped, and characterized in our laboratory (Table 1). While the majority of cultures were karyotypically normal diploid population (hNPC^dip^ in Fig. 1A and B), some sub-cultures of five hNPC lines (G001, G002, G010, M031, and M046) displayed a complete trisomy of chromosome 7 (hNPC^+7^) after 9–15 weeks in culture (24% of the 21 lines tested) confirmed using both G-banding and FISH (Table 1, Figure 1C and D, respectively). Furthermore, in the M031 line a trisomy of chromosome 19 (hNPC^+19^) appeared after 15–25 weeks in culture (5% of the 21 lines tested) (Table 1, Figure 1E and F). Two of the hNPC lines were obtained from fetal brain tissue of Down's syndrome patients that present with a trisomy of chromosome 21, however, these lines did not show trisomy 7 or 19. The only other unbalanced karyotype was observed in one line with an intercalary deletion of chromosome 13, in band q21 (Table 1), suggesting a unique sensitivity of chromosomes 7 and 19 to trisomy.

**Table 1:** Abnormal findings in cytogenetic and FISH analysis performed on twenty-one independently derived hNPC lines.

No.	hNPC lines	Passage	Karyotype *	Abnormal (%) *	+7 (%) †	+19 (%) †	+7 and +19 (%) †
**1**	G001 VM	p29	NP	NP	Normal	NP	NP
**2**	G002 VM	p29	NP	NP	Normal	NP	NP
**3**	G001 CTX **¶**	p10 – p15 **#**	47,XY,+7	16 – 100	10 – 70	NP	NP
**4**	G002 CTX **¶**	p9 – p18 **#**	47,XY,+7	10 – 100	16 – 100	NP	NP
**5**	G007 CTX	p3 – p4 **#**	NP	NP	Normal	Normal	Normal
**6**	G010 CTX **Δ**	p4 – p20 **#**	46, XY	NP	Normal	Normal	Normal
	G010 CTX **Δ ¶**	p23 – p25 **#**	NP	NP	2 – 3.4%	Normal	Normal
**7**	G016 CTX	p4 – p20 **#**	NP	NP	Normal	Normal	Normal
**8**	K050 CTX	p25	Normal	NP	NP	NP	NP
**9**	K055 CTX	p20	Normal	NP	NP	NP	NP
**10**	K057 CTX	p38	Normal	NP	NP	NP	NP
**11**	M006 CTX	p3 – 21 **#**	Normal	NP	NP	NP	NP
**12**	M007 CTX	p3	Normal	NP	NP	NP	NP
**13**	M024 CTX	p12	Normal	NP	NP	NP	NP
**14**	M031 CTX **Δ**	p4 – p29 **#**	Normal	NP	Normal	Normal	Normal
	M031 CTX^+7^ **Δ ¶**	p14 – p38 **#**	47,XY,+7	5 – 100	3 – 99	Normal	Normal
	M031 CTX^+19^ **Δ ¶**	p19 – p38 **#**	47,XY,+19	5 – 95	Normal	3 –55	Normal
	M031 CTX **Δ ¶**	p29	47,XY,+19	40	NP	NP	NP
	M031 CTX **Δ ¶**	p29	48,XY,+7, +19	5	NP	NP	NP
	M031 CTX **Δ ¶**	p38	NP	NP	93	Normal	5
	M031 CTX **Δ ¶**	p38	NP	NP	34	3	0
**15**	M038 CTX	p20	NP	NP	Normal	Normal	Normal
**16**	M046 CTX **Δ**	p4 – 21 **#**	Normal	NP	NP	NP	NP
	M046 CTX **Δ ¶**	p13	NP	NP	2	Normal	Normal
**17**	M067 CTX	p4	Normal	NP	NP	NP	NP
**18**	M099 mid CTX	p4	NP	NP	Normal	Normal	Normal
**19**	M099 OCCCTX	p4	NP	NP	Normal	Normal	Normal
**20**	B001 CTX **^∧^**	p2	47,XY,+21	99	NP	NP	NP
**21**	B003 CTX **^∧^**	p3	47,XX,+21	99	NP	NP	NP

*- Cytogenetic G-band karyotyping results with the percent abnormal based on analysis of 20 metaphase cells, **†** - FISH analysis results showing the percent of cells displaying either trisomy 7 (+7) or trisomy 19 (+19) based on counting signals in at least 200 nuclei. Normal results from a cytogenetic or FISH study are stated. Tests that were not performed are represented by “NP” in the relevant column. Whenever an aberration was detected, the absolute number or the range of per cent abnormal cells from passage to passage is indicated.

**Δ -** The results for multiple passages from one line or its derivatives tested are listed in different rows.

# **-** The passage range in a row indicates testing of the line or its derivative at different passages.

**¶ -** Five out of 21 lines were identified as susceptible to +7 and only M031 CTX was identified with +19.

**∧**- B001 and B003 CTX lines were obtained from Down's syndrome patients that present with a trisomy 21 (+21).

Once detected in a sub culture, the frequency of hNPC^+7^ or hNPC^+19^ occurring cells increased over subsequent passages and entirely predominated within ten to fifteen weeks of first detection suggesting a selective advantage of the trisomy cells (Table S1). Although these trisomies were seen in some thawed cell lines, other lines derived from the parental M031^dip^ line maintained their normal karyotype for over fifty weeks in culture, after which they senesced (data not shown). Thus, the process of trisomy formation between different hNPC lines or selection of rare trisomy cells within the culture appears stochastic. Subsequent studies in the current report were performed with the karyotypicallly normal diploid M031 line (M031^dip^) and its trisomy derivatives, M031^+7^ and M031^+19^.

### Trisomy hNPCs display faster growth kinetics and a proliferative advantage

In order to determine the effect of these trisomies on cell growth and survival, we isolated normal hNPC^dip^, 100% hNPC^+7^, and 95% hNPC^+19^ sub cultures at passage 25 from the M031 cell line. Based on morphological appearance, the trisomy cultures grown as neurospheres did not differ from normal diploid line (Figure S1A). Both showed normal numbers of filopodia at their outer edges and could be efficiently expanded, dissociated and plated. Subsequently, volumetric measurements of single spheres were used to assess the growth rates of the three lines. Neurospheres from M031^+7^ and M031^+19^ cultured under standard conditions had ∼100% and 60% greater increase in neurosphere volume, respectively, than the diploid line ().

**Figure 2:**
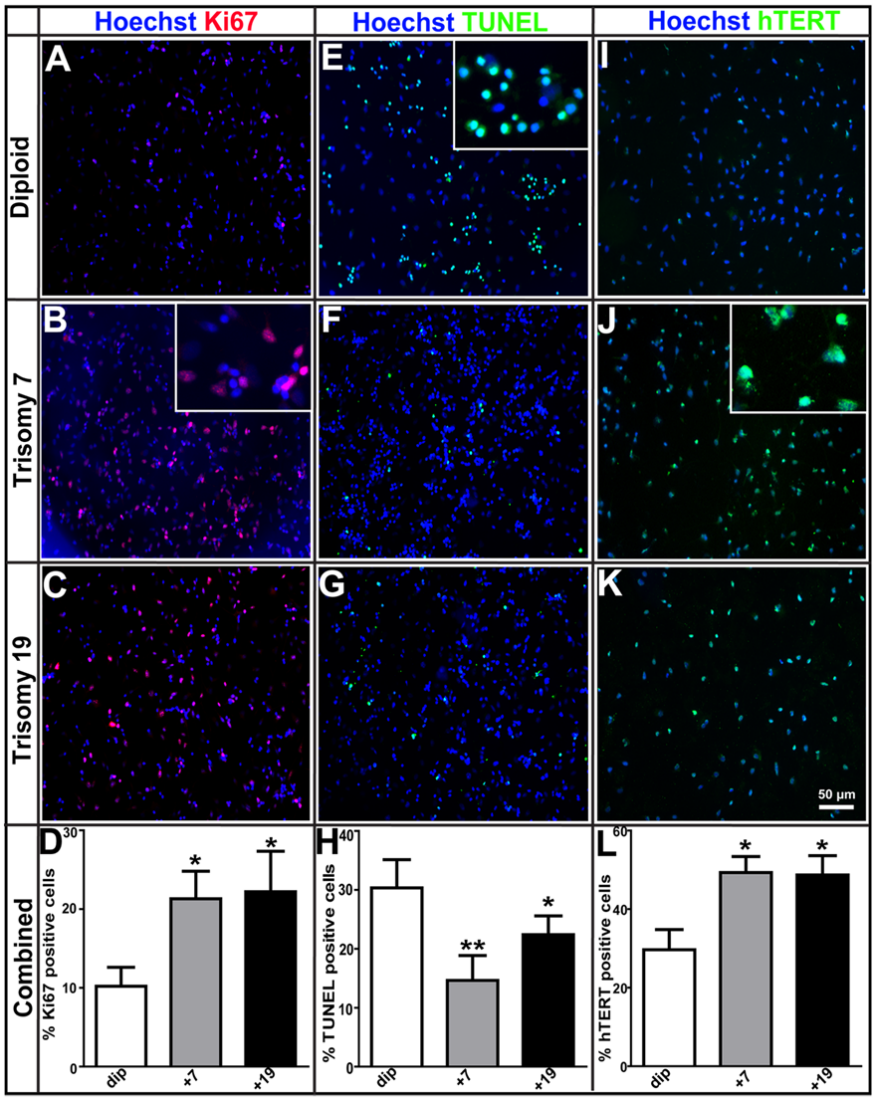
Growth and proliferative advantage of M031^+7^ and M031^+19^ over M031^dip^ CTX hNPCs. (A–D) Immunocytochemistry for Ki67 protein, a cell-cycle associated proliferation marker, shows significantly more Ki67 positive cells in both M031^+7^(21%) and M031^+19^ (22%) compared to the M031^dip^ line (10%). (E–H) Cell death analysis using TUNEL staining shows that compared to the M031^dip^ control (31%), there was a significant decline in the number TUNEL positive cells in the M031^+7^ (13%) and M031^+19^ (22%) hNPCs. (I–L) Immunocytochemistry for the catalytic subunit of telomerase (hTERT) exhibits significant increase in the number of hTERT positive cells in M031^+7^ (49%) and M031^+19^ (48%), when compared to M031^dip^ controls (∼31%). p value: ** <0.01 and * <0.05. The magnified images for (B) Ki67, (E) TUNEL, and (J) hTERT immunostaining are shown in insets. Images are representative of one of three independent experiments with similar results. The data in the graphs are averaged over three independent experiments with mean and SEM values.

To determine whether the faster growth kinetics observed in the cultures with trisomy was due to increased cell proliferation or inhibition of cell death, we performed both proliferation and cell death assays. The Ki67 protein is a well-known cellular marker strictly associated with cell proliferation. Neurospheres from the three lines were dissociated as single cells and plated down on glass coverslips for 2 h prior to fixation and staining with Ki67 (Figure 2A–C). There were significantly more Ki67 positive cells in both M031^+7^ (21%) and M031^+19^ (22%) compared to the M031^dip^ control parental line (10%) (Figure 2D).

We next visualized cells exhibiting typical morphological features of apoptosis such as DNA fragmentation, chromatin condensation and pyknotic nuclei using TUNEL staining (Figure 2E–G). This showed a decreased number of TUNEL positive cells, (13% and 22% in M031^+7^ and M031^+19^, respectively) compared to the M031^dip^ line (30%) (Figure 2H). These results suggest that both increased proliferation and decreased cell death contribute to the increased survival and growth of the hNPC^+7^ and hNPC^+19^ lines.

Limited replicative capacity is a consistent characteristic of somatic cells during *in vitro* passaging, ultimately resulting in senescence mainly associated with telomere erosion [24]. Human telomerase reverse transcriptase (hTERT) expression can inhibit telomere erosion and has powerful effects including the induction of senescence-associated genes [25]–[27]. To determine whether telomerase was altered in the aneuploid hNPCs to explain extended growth, we quantified telomerase expression by immunocytochemistry (Figure 2I–K). There was a significant increase in the number of hTERT positive cells in M031^+7^ and M031^+19^ hNPCs after acute platedown to ∼49% and 48%, respectively, when compared to M031^dip^ cells (∼31%) (Figure 2L). However, even with higher telomerase expression the trisomy hNPCs entered a senescence phase between 50 and 70 weeks *in vitro* (data not shown). These data suggest that while increases in hTERT may enhance proliferation and survival rates, they may not delay eventual senescence of the cells as we have shown previously for diploid hNPCs [19].

### Trisomy hNPCs demonstrate enhanced survival and neurogenesis following differentiation

To determine whether the trisomy hNPCs have a survival advantage during differentiation, the percent of Ki67 and TUNEL positive cells were compared with M031^dip^ controls at similar passages. Interestingly, the survival of trisomy hNPCs (22–30% Ki67+ and 18–26% TUNEL+) upon differentiation was significantly increased when compared to M031^dip^ controls (12% Ki67+ and 24–38% TUNEL+) at similar passages (Figure S2).

Next, we assessed the rate of neurogenesis by dissociating neurospheres and plating them on laminin coated coverslips in mitogen-free media and establishing the number of differentiating neurons and astrocytes. The M031^+7^ and M031^+19^ lines generated significantly more β_III_-tubulin positive neurons compared to M031^dip^ controls following one day, one week and two weeks of differentiation (Figure S3). In addition, GFAP positive astrocytes decreased proportionately in M031^+7^ and M031^+19^ (45–72%) in comparison to M031^dip^ controls (70–90%). Thus, the trisomies led to increased survival and neurogenesis with decreased gliogensis following differentiation of hNPCs.

### EGFR family is over-expressed in trisomy hNPCs

**Figure 3:**
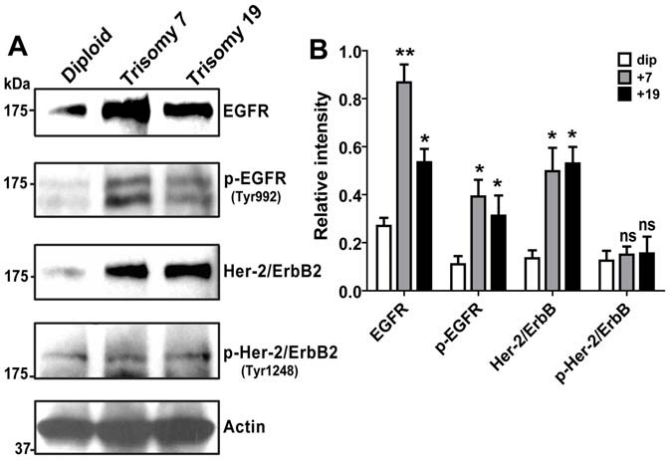
EGF signaling is important in survival advantage of trisomy hNPCs. (A) Western blot analysis illustrated that M031^+7^ and M031^+19^ lines overexpress EGFR family proteins resulting in increased activation of phosphorylated EGFR (Tyr992). (B) Desnitometry quantification of scanned bands using ImageJ 1.17 software displays ∼2 to 3-fold increase in EGFR protein levels and 3 to 4-fold increase in Her-2 protein levels in trisomy hNPCs. p value: ** <0.01,* <0.05, and ns = not significant. Immunoblotting images are representative of one of three independent experiments with similar results. The data in the graph is averaged over three independent experiments with mean and SEM values.

In order to examine the molecular mechanisms underlying the differences between diploid and trisomy hNPCs we compared the expression of signal transduction genes using Affymetrix GeneChip DNA microarray analysis comparison of M031^dip^ and M031^+7^ lines. This experimental design allowed us to reduce the statistical impact of line to line variations. Of the total genome, microarray comparison revealed 1,154 genes up-regulated more than 1.5 fold and 462 genes down regulated. Only 105 genes were up-regulated and 16 were down-regulated more than 1.5 fold of the total 1,150 genes on chromosome 7. Interestingly, the expression of the epidermal growth factor receptor (EGFR) gene increased 1.7 fold (Table S2). To confirm the changes in EGFR expression, Western blot analysis performed on cell lysates from M031^dip, +7, and +19^ lines demonstrated that M031^+7^ and M031^+19^ lines over-express EGF receptor family proteins, including human epidermal growth factor receptor 2 (Her-2/neu/ErbB2), resulting in increased activation of phosphorylated EGFR (Tyr992) (Fig. 3A). Densitometric quantification of the protein bands supports the GeneChip EGFR mRNA over-expression data (Figure 3B).

### Exogenous EGF depletion may favor survival of trisomy hNPCs *in vitro*

**Figure 4:**
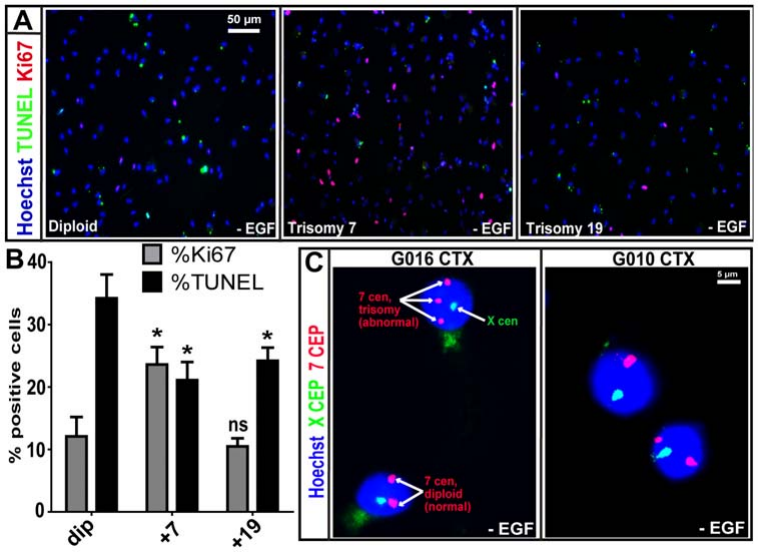
EGF independence in M031^+7^ hNPCs and EGF deprivation to a normal G016 CTX line leads to trisomy of chromosome 7. (A, B) Following 12-day EGF withdrawal, M031^+7^ line manifests a 2-fold increase in EGF-independent growth evident from an increase in Ki67 positive cells and decrease in TUNEL positive cells by immunocytochemistry. p value:,* <0.05, and ns = not significant. Images in A are representative of one of three independent experiments with similar results. The graph in B is averaged over three independent experiments with mean and SEM values. (C) FISH analysis for control centromeric chromosome enumerating probes (CEP) X (green) and CEP 7 (red) shows that EGF depletion for four weeks from the hNPC expansion media results in stress-associated trisomy of chromosome 7 in G016 hNPCs, as indicated by the three red spots in the nucleus. Normal diploid cells display two red spots. 7% (14/200)±1% of the cell population had trisomy 7 after nuclei were counted. Under similar conditions the G010 CTX line never displayed any trisomy 7. This experiment was repeated two times, data was averaged over those experiments and represented by mean and SEM values.

To address the possibility that M031^+7^ cells may manifest EGF-independent growth characteristics, we omitted EGF from the hNPC expansion media supplemented only with LIF. Following 12-day EGF withdrawal, M031^+7^ showed a 2-fold increase in EGF-independent growth when compared to M031^dip^ as revealed by an increase of 9% in Ki67 positive cells and a 10% decrease in TUNEL positive cells (Figure 4A and B), which may likely be a result of EGF receptor over-expression in the M031^+7^ cells.

Subsequently, we investigated whether EGF deprivation may lead to the emergence of trisomy 7 or other associated abnormalities [28], [29]. Two cortical hNPC lines, G010 and G016, were used. At early passages these cells were karyotypically normal and had never previously been cryo-preserved (Figure 4C). EGF was removed from the media for four weeks and then added back to both cultures at which time they were grown for an additional two weeks and processed for FISH analysis. Interestingly, the G016 line presented an extra copy of a chromosome 7 (7% of the cell population) (Figure 4C), when compared to a sister culture maintained in EGF throughout the experiment. However, the G010 line was karyotypically normal before and after the exogenous EGF depletion (Figure 4C). This data shows that in one hNPC line, stress through removal of exogenous EGF either triggers a trisomy 7 or selects for rare trisomy 7 cell(s) within the culture. In number of well-controlled experiments we attempted to induce trisomy in hNPCs using various acute or chronic cell culture stressors including, sub-optimal cryopreservation and passaging techniques, however, we were never able observe a trisomy in those hNPCs (data not shown).

### Trisomy hNPCs survive better in rat brain xenografts but do not form tumors

**Figure 5:**
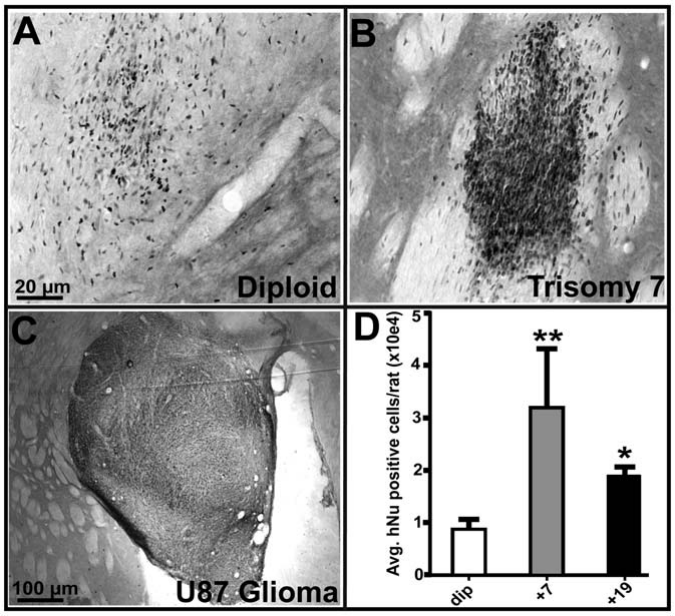
Rat brain transplants of M031^+7^ and M031^+19^ hNPCs show improved survival, however, do not induce tumorigenesis. (A–C) Immunohistochemical stain for human nuclear antigen (hNu) of M031^dip^ hNPCs, transplanted into the striatum of unlesioned rats in one hemisphere along with (B) M031^+7^ and (C) U87 glioma cells in the corresponding hemisphere. M031^+7^ (and M031^+19^; not shown here) hNPCs showed no signs of invasiveness as compared to the positive control U87 glioma cells. (D) The relative proportions of surviving hNu positive cells 6-weeks post-transplantation were noted by visual observation and scored. The overall survival of M031^+7^ and M031^+19^ compared to M031^dip^ control significantly increased. p value is ** = 0.007, * = 0.01.

In order to test whether a trisomy line may induce tumor formation *in vivo*, the M031^+7, and +19^ hNPCs were transplanted into the striatum of adult rats in one hemisphere along with M031^dip^ controls in the contralateral hemisphere. The U87 glioma line (similar to some brain tumor stem cell lines exhibiting typical neoplastic karyotype: Figure S4) was used as a positive control for malignant tumor formation. After 6 weeks, the animals were sacrificed and the numbers of surviving cells were then counted in the striatum using the human specific nuclear marker (hNu). The M031^dip^ cells had a typical transplant survival as we have observed in previous studies (Figure 5A) [18], [30], [31]. The overall survival of hNPCs was significantly increased in the trisomy 7 and 19 groups when compared to diploid controls (Figure 5B and D). However, there was no sign of perivascular cuffing or other cellular overgrowth suggestive of tumor formation (Figure 5B). We utilized the U87 cells, a highly aggressive astrocytoma line, as controls showing that tumors can form in the rat model. In stark contrast to the hNPC lines, U87 cells formed tumors of a progressively enlarging, well-defined cell mass consisting of highly packed undifferentiated small rounded single mitotic elements (Figure 5C).

## Discussion

In the present study, we illustrate that while many hNPC lines derived from different fetal sources are karyotypically normal, ∼24% of our lines showed a trisomy of chromosome 7 and only one line displayed trisomy 19. These trisomies appear to occur stochastically between different hNPC lines. However, no other trisomies were detected in other lines despite extensive karyotyping. Therefore, provided culture conditions and karyotyping is carefully monitored, these cells still provide a safe source of neural tissue for researchers and future clinical trials. Interestingly, we also demonstrated that the stress of EGF withdrawal can lead to trisomy 7 cells emerging from karyotypically normal diploid cultures, suggesting that maintaining optimal culture conditions may be crucial for long term growth of the cells.

The occurrence of cytogenetic aberrations is common in dividing cells, but most are not viable. However, survival of cells with specific chromosome changes, and their ability to replace the normal diploid cells in culture, implies that these changes impart a growth advantage to those cells in which they arise. These cells are termed “culture-adapted” since they have been selected for improved growth in culture [2], [32]. Cultured hESCs have been reported to acquire genetic and epigenetic changes that make them vulnerable to transformation and neoplastic transformation even without harboring any chromosomal abnormalities [33]. Selection for multiple recurrent cytogenetic changes may lead to uncontrolled cell division and morphological alterations, thus converting a normal cell to a cancer cell (i.e. transformation). Therefore, according to the multi hit model of cancer early cytogenetic changes like trisomy 7 or 19 that lead to a growth advantage may be the “first hit” required for progression to transformation, ultimately resulting in tissue-specific tumors. For example, trisomies 12 and 17 are characteristic findings in human germ cell tumors, while trisomies 8 and 11 are common changes in mouse tumors [2], [6], [7], [34]. Interestingly, trisomy 7 is a characteristic finding in glioblastoma [35], [36] and trisomy 19 is characteristic of ependymoma [37]. Thus, just as hESC trisomies mirror changes in germ cell tumors [34], [38], trisomies of 7 and 19 may mirror chromosome changes seen in tumors of neural lineage. Future experiments in our laboratory will test this hypothesis.

The trisomy hNPCs displayed faster population doubling in culture than the diploid hNPCs, partly due to increased cell proliferation (Fig. 2A–D) and inhibition of cell death (Fig. 2E–H). Human telomerase catalytic subunit (hTERT) expression is crucial for induction of senescence-associated genes and eventual immortalization. Even though the trisomy hNPCs display higher telomerase expression and extended growth in culture, they are not immortalized as they enter a senescence phase between 50 and 70 weeks. It is known that senescence can be initiated by the shortening of telomeres (replicative senescence) or by other endogenous and exogenous acute and chronic stress signals in a process termed as STASIS (STress or Aberrant Signaling Induced Senescence) [39]. Growth inhibitory genes can be activated in cell culture and *in vivo* due to STASIS or culture shock. STASIS works through a different mechanism than telomere-based replicative senescence. While cells undergoing replicative senescence can be immortalized by expression of hTERT to maintain telomere homeostasis, this does not occur in cells undergoing growth arrest due to STASIS [39].

Interestingly, we observed an increase in EGFR expression and activation (Tyr992 phosphorylation) in the trisomy 7 and 19 hNPCs. The SH2 domain of PLCγ binds at phospho-Tyr992, resulting in activation of PLCγ-mediated downstream signaling [40]. The presence of EGFR gene on chromosome 7 may reconcile the selective proliferative advantage of the hNPC^+7^ cells. Additonally, important genes that regulate cell cycle, proliferation and apoptosis including, JunD (a c-Jun proto-oncogene family transcription factor), mitogen-activated protein kinase kinase 2 (MAP2K2), Janus Kinase 3 (JAK3, and transforming growth factor-beta 1 (TGFβ1) are located on chromosome 19 [41]. It is possible that some of these critical regulators of cell proliferation up-regulated in the trisomy 19 cell lines play an important role in paracrine/autocrine signaling resulting in EGFR overexpression and autoactivation.

In the postnatal subventricular zone (SVZ) neural stem cells (NSCs) give rise to transit-amplifying precursors (TAPs) expressing high levels of EGFR that in turn generate neuroblasts. Recently, it has been shown that distal-less (DLX)2 homeobox transcription factor and EGFR signaling interacts at multiple levels to coordinate neurogenesis and proliferation in the postnatal SVZ by promoting the lineage transition from NSCs to TAPs [42]. Other studies have also elucidated the importance of EGFR expression and signaling during regulation of neurogenesis [43]–[45]. Since both the trisomy hNPC lines overexpress EGFR and have increased EGFR activation in culture, it is likely that this may lead to increased generation of neuroblasts and subsequent increase in neuron production upon differentiation when compared to the diploid line. We have also shown that hNPC neurospheres grown in high concentrations of EGF grow faster and have greater neurogenesis [46].

A major role of EGFR in the malignant transformation is emphasized by loss of EGF sensitivity and acquisition of an extra chromosome 7p harboring the EGFR gene, as a consequence of EGF deprivation [28]. There is recent indication that neural stem cells within subventricular zone (SVZ) may give rise to tumors like GBM's and suggestions that telencephalic proliferative regions of the mammalian brain, including the embryonic cerebral cortex and postnatal SVZ may contain a population of aneuploid cells [20], [21], [47], [48]. Approximately, 50–75% of glioblastoma's (GBM's) exhibit EGFR gene amplification and trisomy/polysomy of chromosome 7 in addition to other chromosomal aberrations [35], [36]. Additionally, a newly recognized subset of trisomy 19 ependymal tumors that are supratentorial WHO grade III tumors of the young has been reported [37].

Cancer arises from accumulations of genetic changes in pathways involved in cell cycle, cell proliferation, apoptosis, angiogenesis and interaction with extracellular matrix. This usually leads to an abnormal karyotype as shown for some BTSCs (Figure S4). The trisomy 7 or 19 cultures described here did not display a cancer phenotype, nor did they produce malignant tumors upon transplantation into immunosuppressed rats. However, we cannot rule out that a trisomy may confer a vulnerability of the cells to subsequent malignant changes under appropriate stress conditions. Given that acquisition of a trisomy is the most frequent karyotypic change found in stem cells, it is possible that these cells are susceptible to errors in proper chromatid separation during mitosis. For example, the G2 decatenation check point responsible for delaying entry into mitosis if the chromosomes have not been sufficiently untangled or decatenated has been reported to be highly inefficient in mouse ESCs [49]. Defects at the mitotic spindle checkpoint that ensures proper sister chromatid alignment before their separation has also been reported to cause aneuploidy if segregation occurs while chromatids are attached to a single spindle [50]. Thus the emergence of clonal populations of karyotypically abnormal stem cells may involve mitotic errors followed by selection for those changes that are most adaptive for those cells in their culture environment. Additionally, hESCs with trisomies demonstrate enhanced neurotrophin signaling, which may provide a selective advantage under suboptimal culture conditions [51]. This suggests that recurrent aneuploidies in cultured cells may be driven by stresses in the immediate cell environment (e.g. media composition, oxygen tension, subculture methods, etc), similar to our observation in this report that stress of EGF deprivation may be a trigger for trisomy 7 (Figure 4C). Alternatively, EGF withdrawal may be selecting for rare cell(s) with trisomy 7 that subsequently due to a selective advantage dominate the culture population. More studies are required to define exactly what causes trisomies in stem cell culture models.

There are different methods being developed for isolating and expanding hNPC cultures by academic labs and commercial companies [8], [9], [52], [53]. StemCells Inc. performs direct isolation of fetal brain tissue-derived hNPCs based on fluorescence-activated cell sorting (FACS) of CD133(+) cells (cell surface marker), subsequent expansion as neurospheres and transplantation of early passage cultures in a clinical trial for Batten's disease [52]–[54]. This company has not reported trisomies within their culture system, although they do not maintain the cells for more than 10 weeks prior to transplantation. Other groups grow fetal and hES-derived NPCs in adherent monolayer culture in EGF and FGF-2 and has shown that they maintain a diploid karyotype [55], [56]. It is unclear whether our method of maintaining cells in EGF and LIF combined with neurosphere chopping rather than mechanical or enzymatic dissociation used in other methods results in a differential rates of trisomy occurrence, or whether trisomies observed in other systems have not been reported. Other protocols are also being developed for transitioning hES-derived NPCs into clinical trials [56]–[59]. In particular, Geron Corp. has recently received FDA clearance for transplanting hES-derived oligodendrocyte neural progenitors into acute spinal cord injury patients [58]–[60]. Again, it is not clear if these methods of hNPC expansion result in aneuploidies in some cultures, however, careful monitoring for the development of trisomies will be required.

In conclusion, our results suggest that microenvironmental cues are powerful factors in the selection of specific hNPC aneuploidies. We detected frequent chromosomal changes in 5 hNPC cell lines, with trisomy of chromosome 7 being the most common. Even though the M031 trisomy hNPCs have high telomerase levels combined with increased proliferation rates and lowered apoptosis, the cells eventually undergo replicative senescence *in vitro* just as other hNPCs [61]. They do not form neoplastic overgrowths following transplantation into the adult rat brain, although, they appear to have improved survival rates compared to normal diploid lines. Furthermore, our previous studies have shown that M031 hNPCs can survive transplantation without displaying any signs of tumor formation into models of photoreceptor degeneration [61], amyotrophic lateral sclerosis [30] and Parkinson's disease [31], supporting the general application and safety of these cells, although we are not entirely certain if some of the hNPC population at the time of transplantation carried chromosome 7 or 19 trisomy. However, a recent report also shows the dangers of grafting an inordinate number of cells and the need for careful monitoring of hNPCs before proceeding to clinical trials [62]. Therefore, regular monitoring of the karyotype of hNPCs (fetal or hESC-derived) will be essential before these cells are used in future clinical trials as trisomy of chromosomes 7 and 19 lessen the reproducibility and reliability of therapeutic outcomes and pose a higher risk of potential tumor formation.

## Materials and Methods

### Ethics statement

Human fetal brain tissue (between 10 and 15 weeks of post-conception) was obtained from the Birth Defects Laboratory at the University of Washington. The method of collection conformed to the guidelines recommended by National Institutes of Health for the collection of such tissues and set out by the University of Washington and the University of Wisconsin, Madison. All the animal care treatment protocols and procedures in the present study were carried out in accordance with the guidelines approved by the University of Wisconsin-Madison Research Animals Resources Center and National Institutes of Health standards of animal care. Institutional Review board approval was obtained for all of these studies.

### Human neural progenitor cell culture

Briefly, hNPCs were prepared from freshly dissected fetal brain cortical tissue was dissociated in 0.1% trypsin and seeded into T75 flasks at a density of 200,000 cells per ml of maintenance medium [Dulbecco's modified Eagle medium (DMEM)/Ham's F12 (7∶3) containing penicillin/streptomycin/amphotericin B (PSA, 1% v/v)] supplemented with B27 (2% v/v; Invitrogen), EGF (20 ng/ml; Sigma-Aldrich), and fibroblast growth factor-2 (FGF-2; 20 ng/ml; R&D Systems) with heparin (5 µg/ml; Sigma). Neurosphere colonies rapidly formed and were passaged every 7–14 days by sectioning neurospheres into 200 µm using an automated tissue chopper as described previously [11], [23]. At 2 weeks after the first passage, the cells were switched to maintenance medium containing N2 supplement (1%; Invitrogen) and 20 ng/ml EGF. After 10 weeks, 10 ng/ml LIF (Chemicon) was added to enhance expansion rates. At this stage, the cultures have generally reached a stable phase of growth where they produce a mixture of neurons and astrocytes upon differentiation as described in detail previously [19], [23]. All cultures were maintained at 37°C in 95% O_2_/5% CO_2_.

### G-banding and FISH

Giemsa (G-banding) stain and FISH using centromeric chromosome enumeration probes (CEP) for chromosomes 7, 3, and X and sub-telomeric probes for chromosome region 19p and 19q were performed on log-phase growth hNPCs according to previously published methods [5].

### Neurosphere growth measurements

Single neurospheres from the three hNPC lines (^dip^, ^+7^, and ^+19^) were placed in a 96-well plate and were measured via Integrated Morphometry Analysis using Metamorph software (Molecular Devices, Dowington, PA). Sphere volume was calculated on day 0 and every second day up to 14 days after plating. Half of the medium was exchanged every second day. Results are plotted as sphere volume.

### Immunocytochemistry and TUNEL assay

At the appropriate time points in culture, plated cells were fixed in paraformaldehyde (PFA, 4% vol/vol) and rinsed in phosphate-buffered saline (PBS). Fixed cultures were blocked in 3% (vol/vol) goat serum with 0.3% (vol/vol) Triton X-100 and incubated with primary antibodies to Ki67 (polyclonal, 1∶1000,) to label proliferating cells, and β-tubulin-III (monoclonal IgG2b, 1∶500; Sigma) and GFAP (polyclonal, 1∶1000; DAKO) to label undifferentiated neural progenitors or differentiating neurons and astrocytes, respectively. After incubation with the primary antibodies, cultures were rinsed in PBS and incubated in either Cy3 or fluorescein-conjugated goat anti-rabbit antibodies. Nuclei were counterstained with Hoechst 33258 (0.5 µg/ml; Sigma) and mounted on glass slides using GelTol ™ Aqueous Mounting Medium (Immunotech). Visualization of hNPCs was performed at 20X magnification (Nikon) and quantification of positive cells was completed using Metamorph Offline software (Universal Imaging Corporation). For TUNEL assay, dead cells were detected through a TdT-mediated dUTP Nick End Labeling (TUNEL) assay and Ki67 staining was later performed. The DeadEnd™ Fluorometric TUNEL System (Promega) kit was used and the manufacturer's instructions were followed. Following completion of TUNEL assay, cells stained using standard immunocytochemical protocols with rabbit anti-Ki67 (1∶750) overnight at 4°C.

### Differentiation studies

Dissociated neurospheres plated onto glass coverslips precoated with poly-ornithine (100 µg/ml) and laminin (10 µg/ml) in wells prefilled with standard plating medium (DMEM/HAMS-F12 supplemented with B27, 2% vol/vol). These conditions allowed for rapid neurosphere adhesion and differentiation under serum-free conditions. All differentiating NPC cultures were maintained in humidified incubators at 37°C (5% CO_2_ in air). Half of the culture medium was replenished every 3 days.

### RNA extraction for Affymetrix GeneChip*s*

Total RNA from diploid and ^+7^ hNPCs that were at approximately passage 20 was extracted using Trizol reagent. Total RNA was further extracted using a chloroform/isopropanol separation method. Finally, RNA was ethanol precipitated and allowed to briefly dry before elution in 33 µl of RNase-free water. The quality of the RNA of each sample was assessed by spectrophotometer readings (optical density 260/280 = 2.0 for each RNA).

### Affymetrix GeneChip sample preparation and analysis

cDNA synthesis was performed for hNPCs using the One-Cycle cDNA Synthesis Kit (Affymetrix, Santa Clara, CA). In brief, double-stranded cDNA was synthesized using a Oligo(dT)24 primer at the 3′ end for priming the firststrand cDNA synthesis by Superscript II reverse transcriptase and the T7 RNA polymerase promoter sequence at the 5′ end. cDNA was purified using the cDNA Sample Cleanup Module provided with the One-Cycle cDNA Synthesis Kit and resuspended in 14 µl Elution Buffer. Biotin-labeled cRNA was synthesized using the IVT Labeling Kit (Affymetrix, Santa Clara, CA) and incubated at 37°C for 16 hours. cRNA was purified with the cRNA Sample Clean-up Module provided with the IVT Labeling Kit and eluted in 21 µl. Spectrophotometric analysis was used to determine the cRNA yield as well as the quality of cRNA (optical density 260/280 = 2.0 for each cRNA). An adjusted cRNA yield was calculated to reflect any carryover of unlabeled total RNA. cRNA was fragmented using the 5X Fragmentation Buffer (Affymetrix, Santa Clara, CA) provided, at a final concentration of 0.5 µg/µl, in order to break down full-length cRNA to 35 to 200 base fragments. Fragmented biotin-labeled cRNA samples were then hybridized to the U133 Plus 2.0 Array at 45°C for 16 hours. Hybridized arrays were washed and double-stained with streptavidin-phycoerthrin using the Fluidics Station 400 (Affymetrix, Santa Clara, CA) as defined by the manufacturer's protocol. RNA extractions were performed on the ^dip^ and ^+7^ hNPC lines and processed separately on GeneChips. Stained U133 Plus 2.0 arrays were scanned at 3 µm resolution using the Affymetrix GeneChip Scanner 3000 at the Gene Expression Center (University of Iowa, Iowa City, IA). GeneChip Operating Software v1.2 (GCOS) was used to analyze the relative abundance of each gene derived from the average difference of intensities. Log transformed data was then analyzed further using the GeneSifter software. Gene expression ratios were generated using M031^dip^ control cells from an RNA extraction as the baseline for comparison with M031^+7^ cells generated from an RNA extraction. Statistical analysis of GeneChip data was conducted using GeneSifter software. Student t-tests were conducted for each data set with only genes with a p value <0.05 being considered in the statistical analysis. The Affymetrix microarray dataset for the samples are deposited with Gene Expression Omnibus (GEO) with accession numbers, GSE18349, GSM458064, and GSM458065.

### Cell transplantation

hNPC neurospheres were prepared for transplantation: U87 glioma control cell line, M031 control neurospheres (^dip^; passage 27), trisomy 7 (^+7^; passage 25),and trisomy 19 (^+19^; passage 26) were collected and the media was removed. Neurospheres were dissociated to single cells by incubation with Accutase for 10 min at 37°C, trypsin inhibitor for 5 min at 37°C, and DNase for 10 min at 37°C, followed by manual dissociation into single cell suspensions. Cells were counted and resuspended at a density of 100,000 cells/µl in a 1∶1 mix of Liebowitz (L-15) media and 0.6% glucose in sterile PBS supplemented with 2% B27. Cell suspensions were maintained on ice during transplantation procedures. Rats were randomly assigned to a cell treatment group and injected unilaterally with 200,000 cells: 6 rats received ^+19^; 6 rats received ^+7^; and 3 rats received U87 cells. All 15 rats received an additional injection of 200,000 ^dip^ cells into the opposite striatum to serve as a control. The striatal injection site was calculated from bregma: AP±0.5; ML±3.3. A 30 g needle attached to a 10 µl Hamilton syringe was lowered to DV -5 and left in place for 3 min. An infusion pump delivered the cells at a rate of 1 µl/min. The needle was left in place for an additional 5 min before being slowly removed. All rats received cyclosporine injections (i.p. 10 mg/kg) 1 day before and every day following transplantation. Two to four weeks following transplantation, rats were perfused with chilled 0.9% saline followed by 4% PFA. Brains were post-fixed for 24 h in 4% PFA followed by 30% sucrose. Brains were then sectioned at 40 µm on a sliding microtome (Leica).

### Immunohistochemistry

Following fixation of brains in 30% sucrose for 48 hours, 40 µm coronal sections were cut on a freezing microtome. Sections were washed in Tris-HCl, incubated in 2N HCl for 30 minutes, and quenched by submerging sections in phosphate-buffered saline (PBS) with 10% hydrogen peroxide in 10% methanol for 30 minutes. To stain for human nuclei (hNu), human nestin and GFAP in grafts, all sections were blocked for one hour in 10% normal horse serum (NHS) at RT followed by a 24 h incubation at 4°C with one or combination of primary antibodies - mouse anti-human nuclei (1∶200, Chemicon) or mouse anti-hNestin (1∶200 need manufacturer) and anti-GFAP in Tris-HCl/0.1% Triton X-100 and BSA. Sections were then incubated in a biotinylated secondary antibody (horse anti-mouse, 1∶200, Vector) followed by Vectastain ABC (Vector). Sections were processed with 3,3-diaminobenzidine (DAB, Sigma) as a chromagen between 3 to 7 min and mounted on glass microscope slides. An ethanol dehydration series was performed (70%, 95%, 100%) and was cleared with CitriSolv. Slides were ultimately coverslipped using GelTol ™ Aqueous Mounting Medium (Immunotech). hNu positive cells were quantified using Metamorph Offline software (Universal Imaging Corporation). The number of human nuclei was determined in every eleventh section throughout the striatum. The total number of human nuclei in the graft was determined by multiplying these counts by the interval.

### Immunoblotting

Cultures were washed twice with PBS and lysed in ice-cold lysis buffer [50 mM Tris-HCl (pH 7.5), 150 mM NaCl, 0.5% Nonidet P-40, 1 mM PMSF, 1 mM NaF, 1 mM DTT and 4 mg/ml complete protease inhibitor cocktail]. Protein concentration was determined in cell lysates using the Bio-Rad protein assay kit. Aliquots of protein were mixed with SDS sample buffer and Western blot analysis performed using standard protocols. Ten or 40 µg protein extracts were denatured in Laemmli sample buffer followed by 5 minutes of boiling and then resolved on a 10% or 8% Tris-glycine gel (Novex, San Diego, CA). After electrophoresis (120 V for 2 hours), the proteins were transferred in 1x transfer buffer (25 mM Tris, 192 mM glycine, 0.1% SDS, and 20% methanol [∼pH 8.4]) to a nitrocellulose membrane (Hybond-ECL; GE Healthcare; Piscataway, NJ), with constant current of 100 mA for 2 or 3 hours. The membranes were then blocked in 5% nonfat dry milk TBS solution for 1 hour at room temperature. The blots were incubated overnight at 4°C with one of the following antibodies: AB19012 (Chemicon International, Temecula, CA), M61403 (Biodesing International, Saco, ME), 1310-01 (Southern Biotechnology, Birmingham, AL), MAB13405 (Chemicon International), MAB3328 (Chemicon International), or AB19078 (Chemicon International). The membranes were washed three times with TBS solution including Tween-20 (TBS-T) incubated with horseradish peroxidase–linked donkey anti-mouse, donkey anti-goat or donkey anti-rabbit antibodies (Santa Cruz Biotechnology, Santa Cruz, CA) for 2 hours at room temperature and then washed four times in TBS-T. Detection of the immunoreactive bands was performed with the ECL chemiluminescene detection kit (GE Healthcare). The bands were scanned and quantitated by densitometry (ImageJ 1.17 software; National Institutes of Health [NIH], Bethesda, MD; available by ftp at zippy.nimh.nih.gov/ or at http://rsb.info.nih.gov/nih-image; developed by Wayne Rasband, National Institutes of Health, Bethesda, MD).

### Statistical analysis

Prizm software (GraphPad software, La Jolla, CA) was used for all statistical analyses. All counting data from immunocyto/histochemical analyses and cell survival in the animals were expressed as mean values ± SEM and analyzed by two-tailed *t*-test or two-way ANOVA with Bonferonni *post hoc* test. Differences were considered significant when *p*<0.05.

## Supporting Information

Figure S1 Growth advantage of M031^+7^ and M031^+19^ over M031^dip^ CTX hNPCs. (A) Morphological comparison of trisomy hNPCs demonstrates significantly greater size in comparison with the wild-type diploid hNPCs. Images are representative of one of three independent experiments with similar results. (B) Volumetric measurements of single neurospheres over two weeks exhibit that M031^+7^ and M031^+19^ had ∼100% and 60% greater increase in neurosphere volume, respectively, than the M031^dip^ line. p value: ** <0.01 and * <0.05. The data in the graph is represented by average of three independent experiments with mean and SEM values.
(6.89 MB TIF)

Figure S2 Increased survival of M031^+7^ and M031^+19^ hNPCs. Upon differentiation for 7 to 14 days the M031^+7^ and MO31^+19^ lines showed significantly greater survival than the M031^dip^ controls, as determined by the percent of Ki67 and TUNEL positive cells. p value: ** <0.01, * <0.05, and ns = not significant. Images are representative of one of three independent experiments with similar results. The data in the graphs are averaged over three independent experiments with mean and SEM values.
(9.88 MB TIF)

Figure S3 Enhanced neurogenesis in M031^+7^ and M031^+19^ hNPCs. Following one, seven, and fourteen days of differentiation, β_III_-tubulin (green) and GFAP (red) immunofluorescence establishes that the M031^+7^ and MO31^+19^ lines generated significantly more β_III_-tubulin positive neurons and proportionately less GFAP positive astrocytes, compared to the M031^dip^ controls. p value: *** <0.001, ** <0.01, * <0.05, and ns = not significant. Images are representative of one of three independent experiments with similar results. The data in the graphs are averaged over three independent experiments with mean and SEM values.
(9.74 MB TIF)

Figure S4 Abnormal karyotype of brain tumor stem cell (BTSC) lines. FISH analysis for chromosome 7 (green) and chromosome 3 (red) using respective chromosome enumerating probes reveals the distinctly abnormal heterogeneous karyotype of three BTSC lines. Nuclei were counterstained with Hoechst dye (blue). FISH staining and analyses were performed in triplicate.
(6.55 MB TIF)

Table S1 Selective advantage of the trisomy hNPCs in culture. Once detected in a sub-culture, the frequency of hNPC^+7^ and ^+19^ occurring cells increased over subsequent passages and predominated within ten to fifteen weeks of first detection. Tests that were not performed are represented by “NP” in the relevant column. Results are representative of at least one of three independent biological samples with similar results.
(0.07 MB DOC)

Table S2 EGFR mRNA is upregulated in hNPC^+7^ cells. Affymetrix GeneChip Microarray comparison of M031^dip^ and M031^+7^ lines revealed that 105 genes were up-regulated and 16 were down-regulated more than 1.5 fold on chromosome 7. Interestingly, expression of the EGFR gene increased 1.7-fold. Gene expression ratios were generated using M031^dip^ control cells from an RNA extraction as the baseline for comparison with M031^+7^ cells generated from an RNA extraction.
(0.25 MB DOC)
