# Supplementary figures and images for: Chromosome 7 and 19 Trisomy in Cultured Human Neural Progenitor Cells
Source: PLoS One. 2009 Oct 29;4(10):e7630. doi: 10.1371/journal.pone.0007630 (PMC2765070; doi:10.1371/journal.pone.0007630)

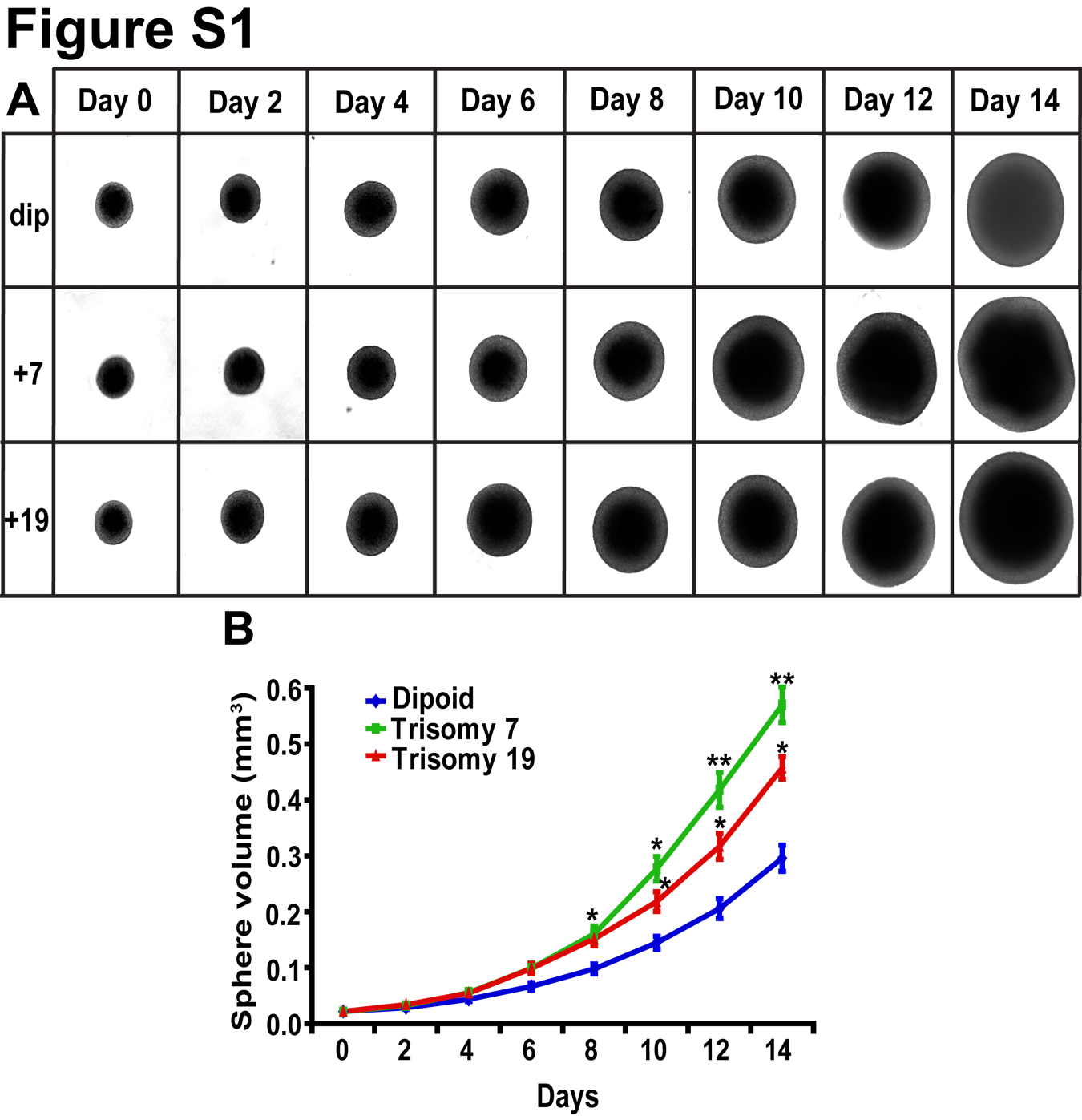

Supplement: Figure S1 — Growth advantage of M031+7 and M031+19 over M031dip CTX hNPCs. (A) Morphological comparison of trisomy hNPCs demonstrates significantly greater size in comparison with the wild-type diploid hNPCs. Images are representative of one of three independent experiments with similar results. (B) Volumetric measurements of single neurospheres over two weeks exhibit that M031+7 and M031+19 had ∼100% and 60% greater increase in neurosphere volume, respectively, than the M031dip line. p value: ** <0.01 and * <0.05. The data in the graph is represented by average of three independent experiments with mean and SEM values. (6.89 MB TIF) [file pone.0007630.s001.tif]

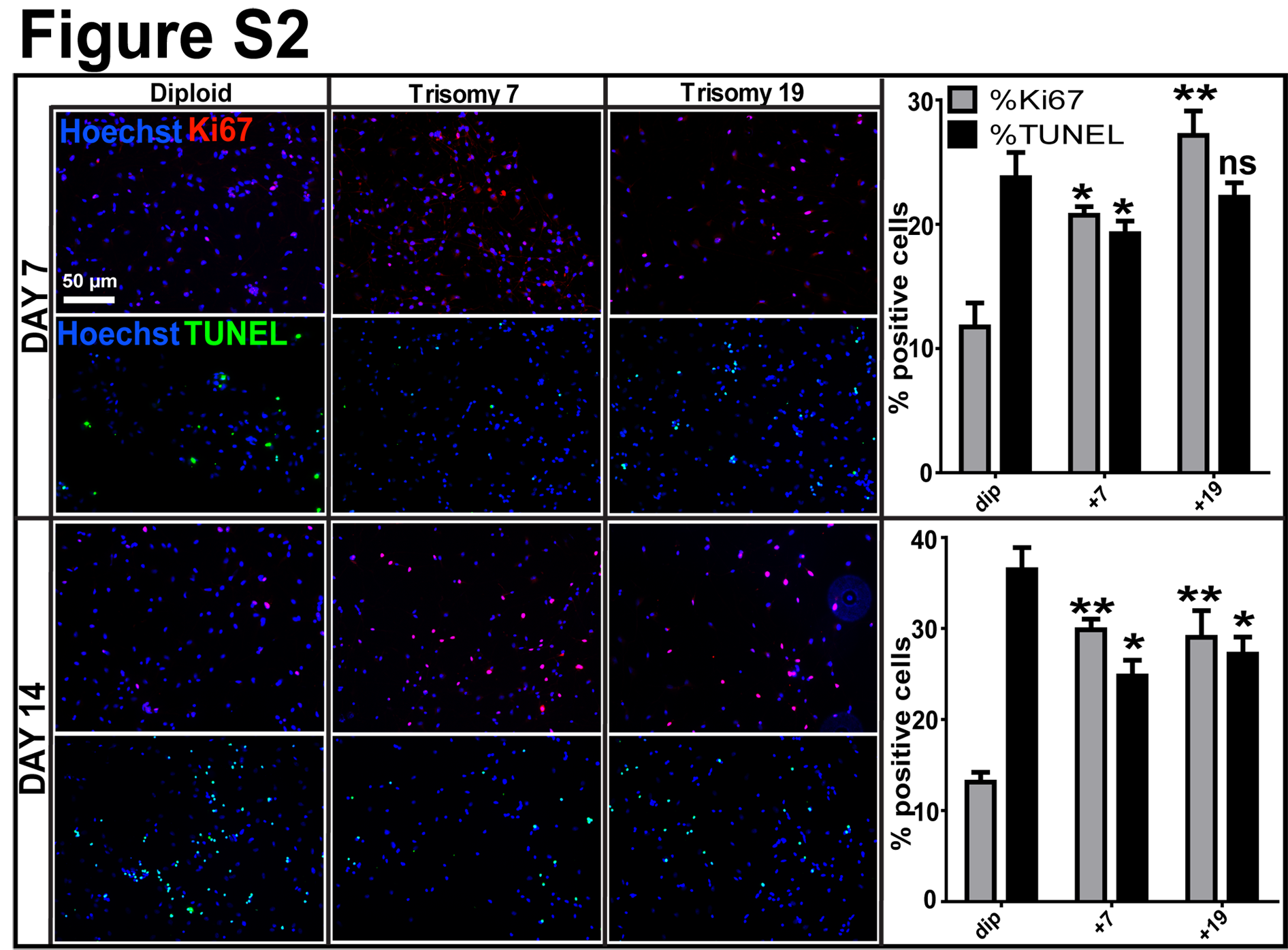

Supplement: Figure S2 — Increased survival of M031+7 and M031+19 hNPCs. Upon differentiation for 7 to 14 days the M031+7 and MO31+19 lines showed significantly greater survival than the M031dip controls, as determined by the percent of Ki67 and TUNEL positive cells. p value: ** <0.01, * <0.05, and ns = not significant. Images are representative of one of three independent experiments with similar results. The data in the graphs are averaged over three independent experiments with mean and SEM values. (9.88 MB TIF) [file pone.0007630.s002.tif]

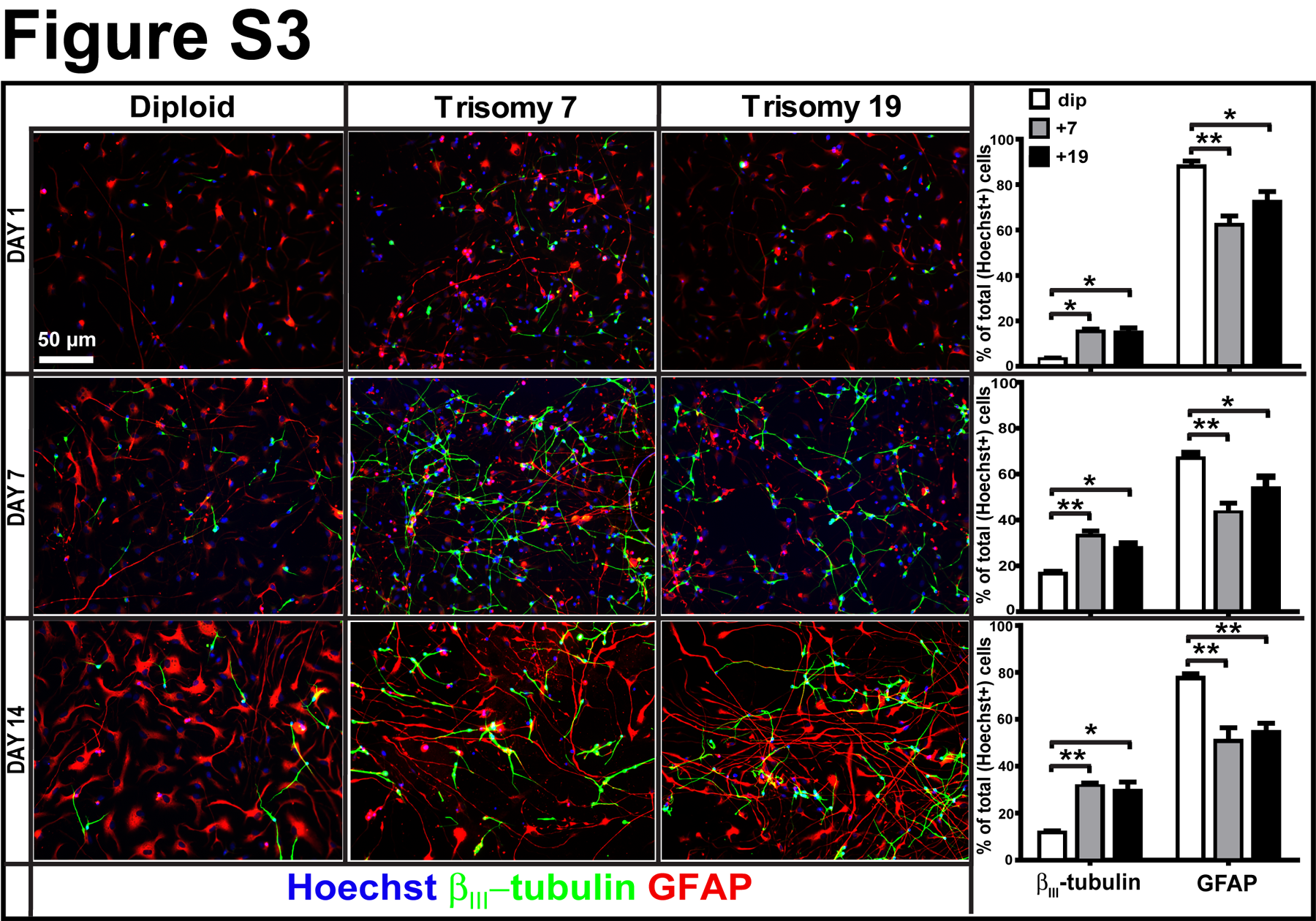

Supplement: Figure S3 — Enhanced neurogenesis in M031+7 and M031+19 hNPCs. Following one, seven, and fourteen days of differentiation, βIII-tubulin (green) and GFAP (red) immunofluorescence establishes that the M031+7 and MO31+19 lines generated significantly more βIII-tubulin positive neurons and proportionately less GFAP positive astrocytes, compared to the M031dip controls. p value: *** <0.001, ** <0.01, * <0.05, and ns = not significant. Images are representative of one of three independent experiments with similar results. The data in the graphs are averaged over three independent experiments with mean and SEM values. (9.74 MB TIF) [file pone.0007630.s003.tif]

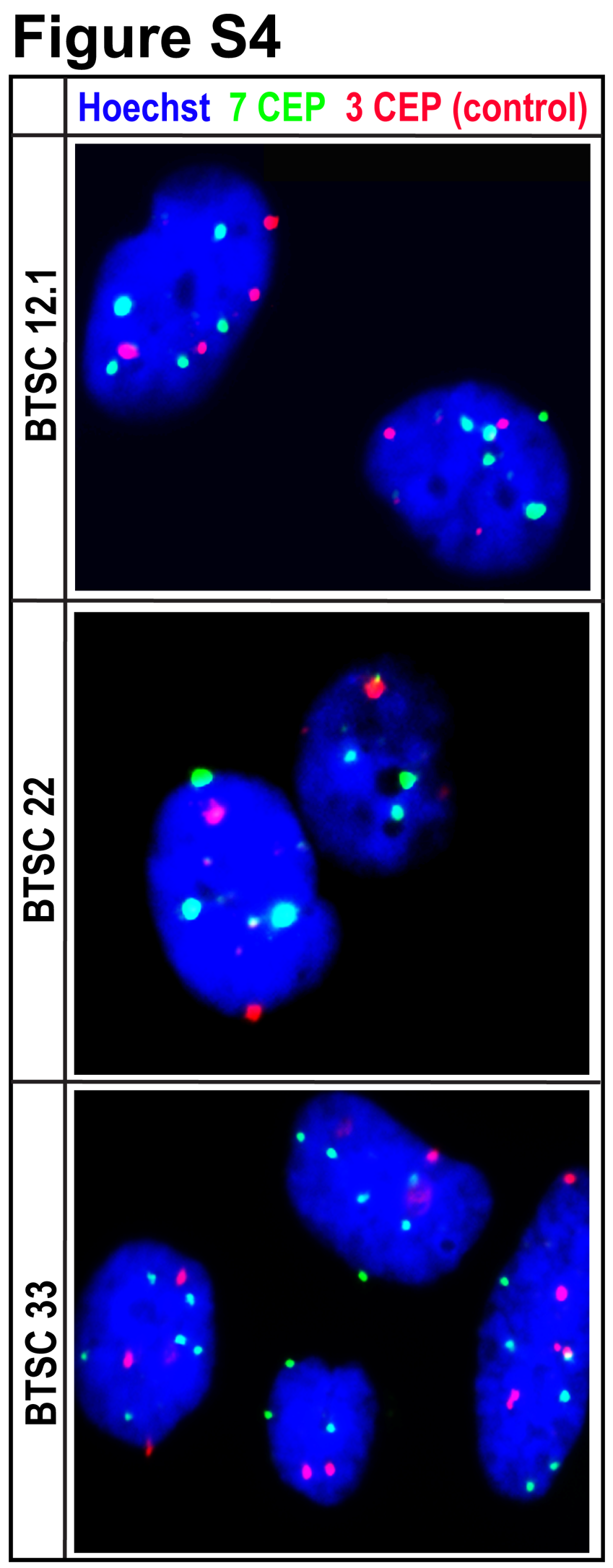

Supplement: Figure S4 — Abnormal karyotype of brain tumor stem cell (BTSC) lines. FISH analysis for chromosome 7 (green) and chromosome 3 (red) using respective chromosome enumerating probes reveals the distinctly abnormal heterogeneous karyotype of three BTSC lines. Nuclei were counterstained with Hoechst dye (blue). FISH staining and analyses were performed in triplicate. (6.55 MB TIF) [file pone.0007630.s004.tif]
