# Supplementary material for: Chromosome 7 and 19 Trisomy in Cultured Human Neural Progenitor Cells
Source: PLoS One. 2009 Oct 29;4(10):e7630. doi: 10.1371/journal.pone.0007630 (PMC2765070; doi:10.1371/journal.pone.0007630)
Supplement: Table S1 — Selective advantage of the trisomy hNPCs in culture. Once detected in a sub-culture, the frequency of hNPC+7 and +19 occurring cells increased over subsequent passages and predominated within ten to fifteen weeks of first detection. Tests that were not performed are represented by “NP” in the relevant column. Results are representative of at least one of three independent biological samples with similar results. (0.07 MB DOC) [file pone.0007630.s005.doc]

**Table S1**

| **hNPC line** | **Sub-line** | **Passage** | **Cytogenetic results** | | **FISH results** | | **DNA fingerprint** |
| --- | --- | --- | --- | --- | --- | --- | --- |
|  |  |  | **Results** | **% abnormal** | **% trisomy 7** | **% trisomy 19** |  |
| **G001 CTX** | --- | 10 | 47,XX,+7 | 16% | 70% | NP | NP |
|  |  | 15 | NP | NP | 16% | Normal | G001 |
| **G002 CTX** | --- | 9 | NP | NP | 46% | Normal | NP |
|  |  | 15 | NP | NP | 100% | Normal | G002 |
|  |  | 18 | 47,XY,+7 | 100% | Normal | Normal | NP |
| **G010 CTX** | --- | 4 | NP | NP | Normal | Normal | NP |
|  |  | 15 | NP | NP | Normal | Normal | NP |
|  |  | 16 | 46,XY | Normal | Normal | Normal | NP |
|  |  | 23 | NP | NP | 2% | Normal | NP |
|  |  | 25 | NP | NP | 3.4% | Normal | NP |
| **M031 CTX** | **M031^+7^** | 14 | 47,XY,+7 | 5% | 4% | NP | NP |
|  |  | 15 | 47,XY,+7 | 10% | 5% | NP | NP |
|  |  | 31 | 47,XY,+7 | 100% | NP | Normal | M031 |
|  |  | 38 | 47,XY,+7 | 100% | NP | Normal | NP |
|  | **M031^+19^** | 19 | 47,XY,+19 | 5% | 4% | N/A | NP |
|  |  | 21 | NP | NP | Normal | 4% | NP |
|  |  | 27 | NP | NP | Normal | 33% | NP |
|  |  | 29 | 47,XY,+19 | 15 – 80%  (7 lines) | Normal | 13 – 55%  (3 lines) | NP |
|  |  | 31 | 47,XY,+19 | 65% | NP | NP | M031 |
|  |  | 38 | 47,XY,+19 | 95% | NP | NP | NP |
