# Supplementary material for: Chromosome 7 and 19 Trisomy in Cultured Human Neural Progenitor Cells
Source: PLoS One. 2009 Oct 29;4(10):e7630. doi: 10.1371/journal.pone.0007630 (PMC2765070; doi:10.1371/journal.pone.0007630)
Supplement: Table S2 — EGFR mRNA is upregulated in hNPC+7 cells. Affymetrix GeneChip Microarray comparison of M031dip and M031+7 lines revealed that 105 genes were up-regulated and 16 were down-regulated more than 1.5 fold on chromosome 7. Interestingly, expression of the EGFR gene increased 1.7-fold. Gene expression ratios were generated using M031dip control cells from an RNA extraction as the baseline for comparison with M031+7 cells generated from an RNA extraction. (0.25 MB DOC) [file pone.0007630.s006.doc]

**Table S2**

| **Gene Name** | **Gene ID** | **M031^dip^** | **M031^+7^** | **Ratio** | **Direction** | **Gene Identifier** |
| --- | --- | --- | --- | --- | --- | --- |
| cell recognition molecule Caspr2 /FL=gb:AF193613.1 gb:NM_014141.1 | CNTNAP2 | 7.7 | 6.4 | 2.4 | **↓** | AU144598 |
| Homo sapiens tetraspan NET-6 protein (NET-6), mRNA. /PROD=tetraspan NET-6 protein /FL=gb:AF120265.1 gb:NM_014399.1 gb:AF100759.1 | TSPAN13 | 9.5 | 8.5 | 2.0 | **↓** | NM_014399 |
| Homo sapiens dynein, cytoplasmic, intermediate polypeptide 1 (DNCI1), mRNA. /PROD=dynein, cytoplasmic, intermediate polypeptide 1 /FL=gb:AF063228.1 gb:NM_004411.1 | DYNC1I1 | 7.9 | 6.8 | 2.0 | **↓** | NM_004411 |
| Homo sapiens cell recognition molecule Caspr2 (KIAA0868), mRNA. /PROD=cell recognition molecule Caspr2 /FL=gb:AF193613.1 gb:NM_014141.1 | CNTNAP2 | 5.5 | 4.6 | 2.0 | **↓** | NM_014141 |
| Homo sapiens hypothetical protein FLJ11808 (FLJ11808), mRNA. /PROD=hypothetical protein FLJ11808 /FL=gb:NM_024728.1 | C7orf10 | 7.0 | 6.1 | 1.8 | **↓** | NM_024728 |
| hypothetical protein DKFZp761D2324 | WBSCR17 | 9.5 | 8.7 | 1.7 | **↓** | AI972623 |
| ESTs | - | 8.8 | 8.0 | 1.7 | **↓** | AA040332 |
| Human growth hormone-dependent insulin-like growth factor-binding protein mRNA, complete cds. /FL=gb:M31159.1 gb:BC000013.1 | IGFBP3 | 6.6 | 5.9 | 1.6 | **↓** | M31159 |
| tissue factor pathway inhibitor 2 /FL=gb:D29992.1 gb:L27624.1 gb:NM_006528.1 gb:BC005330.1 | TFPI2 | 6.9 | 6.2 | 1.6 | **↓** | AL574096 |
| Homo sapiens distal-less homeo box 5 (DLX5), mRNA. /PROD=distal-less homeo box 5 /FL=gb:NM_005221.3 | DLX5 | 10.2 | 9.5 | 1.6 | **↓** | NM_005221 |
| ESTs | - | 11.5 | 10.8 | 1.6 | **↓** | AA129217 |
| KIAA0810 protein | UNC84A | 5.8 | 5.1 | 1.6 | **↓** | BE615699 |
| ESTs | VSTM2A | 4.9 | 4.3 | 1.5 | **↓** | D60436 |
| ESTs | - | 7.0 | 6.4 | 1.5 | **↓** | AI769947 |
| Homo sapiens truncated epidermal growth factor receptor (EGFR) mRNA, partial cds; alternatively spliced. | EGFR | 8.5 | 7.9 | 1.5 | **↓** | AF277897 |
| Homo sapiens actin-related protein 3-beta (ARP3BETA), mRNA. /PROD=actin-related protein 3-beta /FL=gb:NM_020445.1 gb:AF023453.1 | ACTR3B | 8.2 | 7.7 | 1.5 | **↓** | NM_020445 |
| ESTs | DLX6 | 7.2 | 6.6 | 1.5 | **↓** | T65128 |
| ESTs | THSD7A | 3.9 | 5.6 | 3.3 | **­↑** | AW612111 |
| Homo sapiens serine carboxypeptidase vitellogenic-like (LOC54504), mRNA. /PROD=serine carboxypeptidase vitellogenic-like /FL=gb:NM_031311.1 | CPVL | 6.0 | 7.7 | 3.1 | **↑** | NM_031311 |
| PRO1073 protein | GNAI1 | 5.8 | 7.3 | 2.9 | **↑** | AU153866 |
| ESTs, Highly similar to AC005061 1 similar to AAC79150 (H.sapiens) | MGC87042 | 6.5 | 8.1 | 2.9 | **↑** | AW129021 |
| Homo sapiens mRNA; cDNA DKFZp564K1216 (from clone DKFZp564K1216); complete cds. /PROD=hypothetical protein /FL=gb:NM_002069.1 gb:AL049933.1 gb:AF055013.1 | GNAI1 | 5.7 | 7.2 | 2.8 | **↑** | AL049933 |
| ESTs | CDK6 | 6.9 | 8.2 | 2.4 | **↑** | AW192700 |
| Homo sapiens cDNA FLJ20099 fis, clone COL04544. | - | 6.9 | 8.2 | 2.4 | **↑** | AK000106 |
| Homo sapiens cDNA: FLJ22720 fis, clone HSI14320 | HNRNPA2B1 | 7.6 | 8.8 | 2.4 | **↑** | AI963008 |
| KIAA0960 protein | THSD7A | 6.4 | 7.5 | 2.3 | **↑** | BF447246 |
| hypothetical protein | tcag7.1314 | 5.9 | 7.0 | 2.2 | **↑** | AV700415 |
| ESTs | MKLN1 | 5.7 | 6.8 | 2.1 | **↑** | AW505004 |
| Homo sapiens cDNA FLJ13384 fis, clone PLACE1001062, highly similar to Homo sapiens mRNA for lysine-ketoglutarate reductasesaccharopine dehydrogenase. | AASS | 6.8 | 7.9 | 2.1 | **↑** | AK023446 |
| ESTs | - | 8.0 | 9.0 | 2.0 | **↑** | AI800515 |
| Homer, neuronal immediate early gene, 1B | PURB | 10.3 | 11.3 | 2.0 | **↑** | N25931 |
| Homo sapiens cDNA FLJ11581 fis, clone HEMBA1003598 | - | 6.7 | 7.7 | 2.0 | **↑** | AU145003 |
| ESTs | - | 4.5 | 5.5 | 2.0 | **↑** | AI949772 |
| Homer, neuronal immediate early gene, 1B | PURB | 8.4 | 9.4 | 2.0 | **↑** | AV709094 |
| Homo sapiens guanine nucleotide binding protein 11 (GNG11), mRNA. /PROD=guanine nucleotide binding protein 11 /FL=gb:NM_004126.1 gb:U31384.1 | GNG11 | 10.3 | 11.2 | 2.0 | **↑** | NM_004126 |
| Homo sapiens cDNA: FLJ22720 fis, clone HSI14320 | HNRNPA2B1 | 10.0 | 11.0 | 2.0 | **↑** | AI375753 |
| ESTs | - | 6.3 | 7.2 | 2.0 | **↑** | AA682674 |
| myeloidlymphoid or mixed-lineage leukemia 3 /FL=gb:AF264750.2 gb:NM_021230.1 | MLL3 | 8.5 | 9.4 | 1.9 | **↑** | AW137099 |
| transformer-2 alpha (htra-2 alpha) | TRA2A | 7.3 | 8.3 | 1.9 | **↑** | AW978896 |
| Homo sapiens P-glycoprotein (mdr1) mRNA, complete cds. /PROD=P-glycoprotein /FL=gb:AF016535.1 gb:M14758.1 gb:NM_000927.2 | ABCB1 | 7.3 | 8.2 | 1.9 | **↑** | AF016535 |
| ESTs, Moderately similar to ALU5_HUMAN ALU SUBFAMILY SC SEQUENCE CONTAMINATION WARNING ENTRY (H.sapiens) | - | 8.5 | 9.4 | 1.9 | **↑** | AW157450 |
| Homo sapiens cAMP response element-binding protein CRE-BPa (H_GS165L15.1), mRNA. /PROD=cAMP response element-binding protein CRE-BPa /FL=gb:NM_004904.1 gb:L05911.1 | CREB5 | 8.9 | 9.8 | 1.9 | **↑** | NM_004904 |
| Homo sapiens cDNA: FLJ23094 fis, clone LNG07379, highly similar to HST000007 Homo sapiens mRNA full length insert cDNA clone EUROIMAGE 293605. | tcag7.1314 | 5.3 | 6.2 | 1.9 | **↑** | AK026747 |
| ESTs | - | 8.7 | 9.6 | 1.9 | **↑** | AI350995 |
| Homo sapiens Krueppel-related zinc finger protein (H-plk), mRNA. /PROD=Krueppel-related zinc finger protein /FL=gb:NM_015852.1 gb:M55422.1 | ZNF117 | 5.7 | 6.6 | 1.8 | **↑** | NM_015852 |
| DNA directed RNA polymerase II polypeptide J-related gene /FL=gb:NM_145325.1 gb:AF468111.1 | POLR2J4 | 9.8 | 10.7 | 1.8 | **↑** | BQ613856 |
| DNA directed RNA polymerase II polypeptide J-related gene /FL=gb:NM_145325.1 gb:AF468111.1 | POLR2J4 | 8.7 | 9.5 | 1.8 | **↑** | BQ613856 |
| ESTs | GLI3 | 9.5 | 10.3 | 1.8 | **↑** | AW021102 |
| ras homolog gene family, member A | - | 8.8 | 9.7 | 1.8 | **↑** | AA806989 |
| ESTs | CREB5 | 10.1 | 10.9 | 1.8 | **↑** | AI819043 |
| Homo sapiens P-glycoprotein (mdr1) mRNA, complete cds. /PROD=P-glycoprotein /FL=gb:AF016535.1 gb:M14758.1 gb:NM_000927.2 | ABCB1 | 8.4 | 9.2 | 1.8 | **↑** | AF016535 |
| ESTs | - | 6.0 | 6.9 | 1.8 | **↑** | AA832073 |
| ESTs | - | 6.4 | 7.2 | 1.8 | **↑** | BF197705 |
| Homo sapiens clone IMAGE:112574 mRNA sequence. | LOC401321 | 6.4 | 7.2 | 1.8 | **↑** | AF143329 |
| ESTs | - | 9.8 | 10.6 | 1.8 | **↑** | H16409 |
| ESTs | - | 6.8 | 7.6 | 1.8 | **↑** | AA922154 |
| cut (Drosophila)-like 1 (CCAAT displacement protein) | CUX1 | 9.3 | 10.2 | 1.8 | **↑** | BE046521 |
| Human proline rich calmodulin-dependent protein kinase mRNA, complete cds. /PROD=proline rich calmodulin-dependent proteinkinase /FL=gb:U23460.1 | CAMK2B | 6.7 | 7.5 | 1.8 | **↑** | U23460 |
| myeloidlymphoid or mixed-lineage leukemia 3 /FL=gb:AF264750.2 gb:NM_021230.1 | MLL3 | 9.9 | 10.7 | 1.7 | **↑** | BE962679 |
| Homo sapiens calciumcalmodulin-dependent protein kinase II beta 6 subunit (CAMKB) mRNA, complete cds. /PROD=calciumcalmodulin-dependent protein kinase IIbeta 6 subunit /FL=gb:AF081924.1 | CAMK2B | 7.0 | 7.8 | 1.7 | **↑** | AF081924 |
| ESTs | - | 7.5 | 8.2 | 1.7 | **↑** | AV733292 |
| ESTs | CDK6 | 7.4 | 8.2 | 1.7 | **↑** | AW194766 |
| Homo sapiens GLI-Kruppel family member GLI3 (Greig cephalopolysyndactyly syndrome) (GLI3), mRNA. /PROD=GLI-Kruppel family member GLI3 /FL=gb:M57609.1 gb:NM_000168.2 | GLI3 | 8.7 | 9.5 | 1.7 | **↑** | NM_000168 |
| Homo sapiens ATP-binding cassette, sub-family B (MDRTAP), member 4 (ABCB4), transcript variant A, mRNA. /PROD=ATP-binding cassette, subfamily B, member 4,isoform A /FL=gb:NM_000443.2 gb:M23234.1 | ABCB4 | 8.2 | 9.0 | 1.7 | **↑** | NM_000443 |
| Homo sapiens cDNA FLJ20653 fis, clone KAT01739 | CDK6 | 10.7 | 11.5 | 1.7 | **↑** | AW274756 |
| ESTs | - | 5.2 | 6.0 | 1.7 | **↑** | AA760738 |
| Homo sapiens KIAA1079 protein (KIAA1079), mRNA. /PROD=KIAA1079 protein /FL=gb:AB029002.1 gb:NM_014916.1 | LMTK2 | 6.4 | 7.2 | 1.7 | **↑** | NM_014916 |
| Homo sapiens phosphodiesterase 1C, calmodulin-dependent (70kD) (PDE1C), mRNA. /PROD=phosphodiesterase 1C, calmodulin-dependent(70kD) /FL=gb:U40371.1 gb:NM_005020.1 | PDE1C | 7.0 | 7.7 | 1.7 | **↑** | NM_005020 |
| Homo sapiens cDNA FLJ20653 fis, clone KAT01739 | CDK6 | 10.0 | 10.8 | 1.7 | **↑** | AA922068 |
| **epidermal growth factor receptor (avian erythroblastic leukemia viral (v-erb-b) oncogene homolog) /FL=gb:NM_005228.1** | **EGFR** | **10.8** | **11.6** | **1.7** | **↑** | **AW157070** |
| GCN1 (general control of amino-acid synthesis 1, yeast)-like 1 | HIPK2 | 11.3 | 12.0 | 1.7 | **↑** | BF594155 |
| Homo sapiens atrophin-1 interacting protein 1; activin receptor interacting protein 1 (KIAA0705), mRNA. /PROD=atrophin-1 interacting protein 1; activinreceptor interacting protein 1 /FL=gb:NM_012301.1 gb:AF038563.1 | MAGI2 | 6.7 | 7.4 | 1.7 | **↑** | NM_012301 |
| GCN1 (general control of amino-acid synthesis 1, yeast)-like 1 | HIPK2 | 9.8 | 10.6 | 1.7 | **↑** | BF529628 |
| Homo sapiens zinc finger protein (ZFD25) (ZFD25), mRNA. /PROD=zinc finger protein (ZFD25) /FL=gb:NM_016220.1 gb:AB027251.1 | ZNF107 | 8.3 | 9.0 | 1.7 | **↑** | NM_016220 |
| Homo sapiens alpha-aminoadipate semialdehyde synthase mRNA, complete cds. /PROD=alpha-aminoadipate semialdehyde synthase /FL=gb:AF229180.1 | AASS | 7.0 | 7.7 | 1.7 | **↑** | AF229180 |
| myeloidlymphoid or mixed-lineage leukemia 3 /FL=gb:AF264750.2 gb:NM_021230.1 | MLL3 | 9.4 | 10.1 | 1.7 | **↑** | AA121529 |
| Homo sapiens cDNA FLJ35395 fis, clone SKNSH2003064, weakly similar to SPLICEOSOME ASSOCIATED PROTEIN 62. | LOC401321 | 7.2 | 7.9 | 1.7 | **↑** | BI823265 |
| Homo sapiens mRNA; cDNA DKFZp434M0420 (from clone DKFZp434M0420) | tcag7.907 | 7.9 | 8.7 | 1.7 | **↑** | AW006934 |
| Homo sapiens cDNA FLJ11731 fis, clone HEMBA1005411 | KIAA1549 | 7.7 | 8.4 | 1.7 | **↑** | AU145652 |
| GCN1 (general control of amino-acid synthesis 1, yeast)-like 1 | HIPK2 | 11.5 | 12.2 | 1.6 | **↑** | AW300045 |
| Homo sapiens homeodomain-interacting protein kinase 2 (HIPK2), mRNA. /PROD=homeodomain-interacting protein kinase 2 /FL=gb:NM_022740.1 gb:AF208291.1 | HIPK2 | 7.8 | 8.5 | 1.6 | **↑** | NM_022740 |
| Homo sapiens RB-associated KRAB repressor (RBAK), mRNA. /PROD=RB-associated KRAB repressor /FL=gb:NM_021163.2 | RBAK | 5.6 | 6.3 | 1.6 | **↑** | NM_021163 |
| Homo sapiens mRNA; cDNA DKFZp434E0528 (from clone DKFZp434E0528) | - | 8.2 | 8.9 | 1.6 | **↑** | AW242701 |
| Homo sapiens cDNA FLJ13754 fis, clone PLACE3000362. | - | 7.4 | 8.1 | 1.6 | **↑** | AK023816 |
| ESTs | MKLN1 | 9.9 | 10.6 | 1.6 | **↑** | D81987 |
| hypothetical protein PP3501 | KIAA1856 | 10.5 | 11.2 | 1.6 | **↑** | AI936523 |
| ESTs | - | 6.3 | 7.0 | 1.6 | **↑** | AI290919 |
| Homo sapiens cDNA FLJ14081 fis, clone HEMBB1002280. | - | 5.2 | 5.9 | 1.6 | **↑** | BM728567 |
| ESTs | - | 5.3 | 6.0 | 1.6 | **↑** | AW052186 |
| Homo sapiens mRNA for KIAA1758 protein, partial cds. /PROD=KIAA1758 protein | CTTNBP2 | 6.3 | 7.0 | 1.6 | **↑** | AB051545 |
| Homo sapiens cDNA FLJ20653 fis, clone KAT01739 | CDK6 | 11.0 | 11.7 | 1.6 | **↑** | AW051349 |
| ESTs | KIAA1549 | 8.4 | 9.1 | 1.6 | **↑** | AV704797 |
| Homo sapiens cDNA: FLJ21435 fis, clone COL04244 | MKLN1 | 9.7 | 10.3 | 1.6 | **↑** | AW611917 |
| Homo sapiens NEDL1 mRNA for NEDD4-like ubiquitin ligase 1, complete cds. /PROD=NEDD4-like ubiquitin ligase 1 /FL=gb:AB048365.1 | HECW1 | 6.4 | 7.1 | 1.6 | **↑** | AB048365 |
| ESTs | HIPK2 | 11.9 | 12.6 | 1.6 | **↑** | BF218115 |
| Homo sapiens clone 24670 mRNA sequence | - | 8.9 | 9.6 | 1.6 | **↑** | R37104 |
| ESTs | RBAK | 6.7 | 7.4 | 1.6 | **↑** | BE618393 |
| Homo sapiens cDNA: FLJ21670 fis, clone COL09010. | - | 5.3 | 6.0 | 1.6 | **↑** | AK025323 |
| ESTs | - | 8.0 | 8.7 | 1.6 | **↑** | AI268231 |
| Homo sapiens transformationtranscription domain-associated protein (TRRAP), mRNA. /PROD=transformationtranscription domain-associatedprotein /FL=gb:NM_003496.1 gb:AF076974.1 | TRRAP | 9.6 | 10.3 | 1.6 | **↑** | NM_003496 |
| Homo sapiens KIAA0716 gene product (KIAA0716), mRNA. /PROD=KIAA0716 gene product /FL=gb:AB018259.1 gb:NM_014705.1 | DOCK4 | 10.1 | 10.8 | 1.6 | **↑** | NM_014705 |
| ESTs | PPP1R9A | 9.4 | 10.0 | 1.6 | **↑** | AI888150 |
| KIAA1051 protein | PEG10 | 12.1 | 12.8 | 1.6 | **↑** | BE858180 |
| Homo sapiens cDNA FLJ12493 fis, clone NT2RM2001635, moderately similar to NUCLEAR ENVELOPE PORE MEMBRANE PROTEIN POM 121. | POM121 | 9.2 | 9.9 | 1.6 | **↑** | AK022555 |
| Homo sapiens mRNA; cDNA DKFZp586I1420 (from clone DKFZp586I1420); partial cds. /PROD=hypothetical protein | DKFZp586I1420 | 8.2 | 8.8 | 1.6 | **↑** | AL050378 |
| Homo sapiens, clone IMAGE:5396406, mRNA. | - | 5.8 | 6.4 | 1.5 | **↑** | BC038209 |
| Homo sapiens cyclin-dependent kinase 6 (CDK6), mRNA. /PROD=cyclin-dependent kinase 6 /FL=gb:NM_001259.1 | CDK6 | 7.7 | 8.3 | 1.5 | **↑** | NM_001259 |
| Homo sapiens similar to RIKEN cDNA 1200014N16 gene (MGC14289), mRNA. /FL=gb:BC008842.1 gb:NM_080660.1 | ZC3HAV1L | 4.6 | 5.2 | 1.5 | **↑** | NM_080660 |
| Homo sapiens (clone 55) macronuclear mRNA. | MLL3 | 6.2 | 6.8 | 1.5 | **↑** | AI479224 |
| Homo sapiens cDNA FLJ11431 fis, clone HEMBA1001094 | - | 7.4 | 8.0 | 1.5 | **↑** | AI689210 |
| Homo sapiens, retinoic acid receptor responder (tazarotene induced) 2, clone MGC:1544, mRNA, complete cds. /PROD=retinoic acid receptor responder (tazaroteneinduced) 2 /FL=gb:BC000069.1 gb:NM_002889.2 gb:AB015632.1 gb:U77594.1 | RARRES2 | 6.8 | 7.4 | 1.5 | **↑** | BC000069 |
| chromosome 7 open reading frame 3 | NOM1 | 7.5 | 8.2 | 1.5 | **↑** | AI419857 |
| Human truncated epidermal growth factor receptor-like protein precursor mRNA, complete cds. /PROD=truncated epidermal growth factor receptor-likeprotein precursor /FL=gb:U95089.1 | EGFR | 8.7 | 9.3 | 1.5 | **↑** | U95089 |
| ESTs | FLJ25778 | 9.0 | 9.6 | 1.5 | **↑** | AI332346 |
| Homo sapiens calciumcalmodulin-dependent protein kinase II beta subunit mRNA, complete cds. /PROD=calciumcalmodulin-dependent protein kinase IIbeta subunit /FL=gb:AF112472.1 gb:AF078803.1 gb:NM_001220.1 | CAMK2B | 7.8 | 8.4 | 1.5 | **↑** | AF078803 |
| ESTs | - | 8.7 | 9.3 | 1.5 | **↑** | BF707423 |
| ESTs, Moderately similar to CALD_HUMAN CALDESMON (H.sapiens) | CALD1 | 7.1 | 7.7 | 1.5 | **↑** | AA705063 |
| ESTs | - | 6.4 | 7.0 | 1.5 | **↑** | BF002121 |
| ESTs | - | 6.1 | 6.7 | 1.5 | **↑** | BE220224 |
| Homo sapiens AE-binding protein 1 (AEBP1), mRNA. /PROD=adipocyte enhancer binding protein 1 precursor /FL=gb:AF053944.1 gb:NM_001129.2 gb:D86479.1 | AEBP1 | 9.6 | 10.2 | 1.5 | **↑** | NM_001129 |
| ESTs | - | 7.7 | 8.3 | 1.5 | **↑** | AW206440 |
| Homo sapiens cDNA FLJ36460 fis, clone THYMU2014801. | FLJ45482 | 7.5 | 8.1 | 1.5 | **↑** | BQ953917 |
| Homo sapiens cell division cycle 2-like 5 (cholinesterase-related cell division controller) (CDC2L5), mRNA. /PROD=cell division cycle 2-like 5(cholinesterase-related cell division controller) /FL=gb:NM_003718.1 gb:M80629.1 | CDC2L5 | 7.7 | 8.3 | 1.5 | **↑** | NM_003718 |
